# Involvement of Nav 1.8 sodium ion channels in the transduction of mechanical pain in a rodent model of osteoarthritis

**DOI:** 10.1186/ar3553

**Published:** 2012-01-07

**Authors:** Niklas Schuelert, Jason J McDougall

**Affiliations:** 1 Department of Physiology & Pharmacology, University of Calgary, 3330, Hospital Drive NW, Calgary, AB, T2N 4N1, Canada; 2 Departments of Pharmacology & Anaesthesia, Dalhousie University, 5850, College Street, PO Box 15000, Halifax, NS B3H 4R2, Canada

## Abstract

**Introduction:**

A subgroup of voltage gated sodium channels including Nav1.8 are exclusively expressed on small diameter primary afferent neurons and are therefore believed to be integral to the neurotransmission of nociceptive pain. The present study examined whether local application of A-803467, a selective blocker of the Nav 1.8 sodium channel, can reduce nociceptive transmission from the joint in a rodent model of osteoarthritis (OA).

**Methods:**

OA-like changes were induced in male Wistar rats by an intra-articular injection of 3 mg sodium monoiodoacetate (MIA). Joint nociception was measured at day 14 by recording electrophysiologically from knee joint primary afferents in response to non-noxious and noxious rotation of the joint both before and following close intra-arterial injection of A-803467. The effect of Nav1.8 blockade on joint pain perception and secondary allodynia were determined in MIA treated animals by hindlimb incapacitance and von Frey hair algesiometry respectively.

**Results:**

A-803467 significantly reduced the firing rate of joint afferents during noxious rotation of the joint but had no effect during non-noxious rotation. In the pain studies, peripheral injection of A-803467 into OA knees attenuated hindlimb incapacitance and secondary allodynia.

**Conclusions:**

These studies show for the first time that the Nav1.8 sodium channel is part of the molecular machinery involved in mechanotransduction of joint pain. Targeting the Nav1.8 sodium channel on joint nociceptors could therefore be useful for the treatment of OA pain, avoiding the unwanted side effects of non-selective nerve blocks.

## Introduction

Osteoarthritis (OA) is a musculoskeletal disorder in which joint degeneration leads to a loss of mobility and function. OA primarily affects the weight bearing joints (for example knees, hips) and is typified by synovitis and degeneration of the articular cartilage and subchondral bone. The most prominent feature but least understood aspect of OA is joint pain which typically worsens with weight bearing and activity. The clinical diagnosis and treatment of OA pain has proven to be a difficult challenge because of the multitude of complex underlying mechanisms and the fact that different patients show a varied response to the same therapy. The preferred first line treatment for OA pain is non-steroidal anti-inflammatory drugs; however, the beneficial outcome of these drugs is limited and some patients fail to achieve any pain relief at all. Therefore, there is a pressing need to understand the causes and mechanisms of OA pain so that more efficacious targets can be identified to help alleviate these debilitating symptoms. Chronic joint pain originates in the periphery by the sensitization of primary afferent nerve fibers innervating the joint leading to heightened neuronal activity [1-4]. Prolonged bombardment from these primary afferents subsequently sensitizes spinal and supra spinal neurons leading to the experience of persistent pathological pain.

Intra-articular injection of monosodium iodoacetate (MIA) inhibits glyceraldehye-3-phosphate dehydrogenase activity in chondrocytes, resulting in disruption of glycolysis and eventual cell death [5-8]. The progressive loss of chondrocytes results in histological and morphological changes to the articular cartilage, closely resembling those seen in human OA [9]. We have shown previously that joints contain mechanosensitive nerves [10] which become sensitized following MIA-induced joint degeneration [11,12] leading to chronic joint pain [13].

Voltage- gated sodium channels (VGSCs) are fundamental in regulating the excitability of neurons and overexpression of these channels can produce abnormal spontaneous firing patterns which underpin chronic pain [14,15]. There are at least nine different VGSC subtypes in the nervous system, and each subtype can be functionally classified as either tetrodotoxin-sensitive or tetrodotoxin-resistant [16]. Neuronal sodium channel subtypes including Nav1.3, Nav1.7, Nav1.8, and Nav1.9 have been implicated in the processing of nociceptive information [17]. The VGSC Nav1.8 is a tetrodotoxin-resistant sodium channel with a distribution restricted to primary afferent neurons [18,19] and the majority of Nav1.8-containing afferents transmit nociceptive signals to pain processing areas of the spinal cord [20]. Changes in the expression, trafficking and redistribution of Nav1.8 following inflammation or nerve injury are thought to be a major contributor to the sensitization of afferent nerves and the generation of pain [21]. The exclusive distribution of this channel on nociceptive neurons makes it an attractive target for the treatment of pain. Targeting of the Nav1.8 sodium channel with genetic deletions and antisense treatment has confirmed that this channel contributes to normal and pathological nociception [19,22-24]. A-803467 is a potent and selective Nav1.8 sodium channel blocker which has been found to attenuate pain in different rat models of neuropathic and inflammatory pain when given systemically [25-27]. The present study investigated if local intra-arterial administration of A-803467 could reduce the hypersensitivity of nociceptive joint afferents and the resultant joint pain in the rat MIA model of OA.

## Materials and methods

### Animals

Experiments were performed on 44 male Wistar rats (340 to 430 g) which were housed in cages at room temperature (22°C) under a 12:12 hour light/dark cycle with free access to water and food. The animal handling and surgical procedures outlined in this study all received ethical approval from the University of Calgary Animal Care Committee and adhered to the Canadian Council for Animal Care guidelines for the care and use of experimental animals.

### Induction of OA

Forty-four rats were deeply anaesthetised with 2% isoflurane in 100% O2 (1 L/minute) until the flexor withdrawal reflex was abolished. OA was induced by an injection of 3 mg sodium monoiodoacetate (Tocris Bioscience, Missouri USA; 50 μl volume) in 0.9% saline into the joint cavity through the patellar ligament. Animals were allowed to recover for 14 days which has consistently been shown to cause severe end-stage OA in this species [5,7,8].

### Surgical preparation for electrophysiological recordings

Following OA development, animals were deeply anaesthetized with urethane (25% stock solution; 2 g/kg, i.p.) and an acceptable depth of anaesthesia was determined by the absence of the hindpaw withdrawal reflex. Core body temperature was measured by a rectally inserted thermometer and maintained at 37°C. The trachea was cannulated and connected to a Harvard rodent respiratory pump to allow artificial ventilation with 100% O2 (stroke volume: 2.5 ml breath frequency: 60 breaths/min). The left carotid artery was then exposed and cannulated with a fine bore catheter (Portex Fine Bore Tubing, 0.5 mm ID, 1.00 mm OD; SIMS Portex Ltd., Kent, UK) containing heparinized saline (100 units/ml). The cannula was connected to a pressure transducer to allow continuous blood pressure measurement as recorded by a blood pressure monitor (BP-1, World Precision Instruments, Sarasota, FL, USA). An additional catheter containing heparinized saline was placed in the left jugular vein (Portex Fine Bore Tubing, 0.40 mm ID, 0.80 mm OD; SIMS Portex Ltd., Kent, UK) and a single administration of the muscle relaxant gallamine triethiodide (Sigma-Aldrich, Ontario, Canada; 50 mg/kg) was injected to eliminate neural interference arising from the hindlimb musculature. The right saphenous artery was cannulated below the knee joint to permit local close intra-arterial injection of A-803467 to the knee joint. A specialized clamp was fixed to the mid-shaft of the isolated right femur and attached to a stereotaxic frame to prevent movement of the proximal aspect of the rat hindlimb. To apply rotation to the knee joint, the right hindpaw was placed in a shoe-like holder and the hindlimb rotated to standardized torque levels as measured by a force transducer and a torque meter (MVD2510; Hottinger-Baldwin Messtechnik, Darmstadt, Germany). Finally, a longitudinal skin incision was made along the medial aspect of the hindlimb and the skin flaps were fixed to a metal 'O' ring to create a pouch which was filled with warm paraffin oil to prevent tissue desiccation throughout the experiment. The technique used for recording afferent activity from articular nerve fiber from rat knee joints has been described previously [28]. To prevent input from the foot and ankle region, the saphenous nerve was transected distally to the knee joint. The saphenous nerve was then isolated in the inguinal region and cut centrally to prevent the generation of spinally mediated reflexes. The saphenous nerve stump projecting from the knee was placed on a small, black Perspex stage. Under a dissecting microscope, the perineurium was removed and fine neurofilaments were dissected free from the nerve using fine watchmaker forceps. The neural elements were then placed over a platinum electrode to record single afferent fiber activity. To ensure that recorded fibers originate from the knee joint, the receptive field of the fibers was identified by the elicitation of a response to gentle probing of the knee joint with a glass rod with a 1 mm tip. The mechanical threshold of each recorded joint afferent was determined by a gradual increase of torque to the joint until the fiber started to elicit action potentials. Non-noxious and noxious outward movements were then applied to the knee with each movement lasting ten seconds. Non-noxious movement is defined here as being within the normal working range of the joint, while noxious rotation refers to torque occurring outside the normal range but not severe enough to cause soft tissue injury. After three movement cycles, identical test rotations were repeated every two minutes such that each fiber was subjected to a total of 22 movements. The conduction velocity of the nerve fibers (C-fibers < 2 meters/second; Aδ-fibers 2-10 meters/second) was determined by electrical stimulation of the receptive field with a bipolar silver wire electrode (1-10 V, 0.5 msec pulse width). The conduction velocity was calculated by dividing the distance between the receptive field and the electrode by the latency between the stimulus artefact and the evoked action potential. Recordings were made before (control) and after close intra-arterial injection of the Nav 1.8 channel blocker A-803467. The dose of A-803467 administered was 500 ug in 100 ul vehicle (2% dimethyl sulfoxide (DMSO), 1% cremophor, 0.9% saline). This dose has previously been shown to be effective in attenuating evoked pain in inflamed hindpaws [27]. Percentage changes in nerve firing rate before and after administration of A-803467 were calculated. Neuronal activity was recorded by a data acquisition system (CED1401, Cambridge Electronic Design, Cambridge, UK) and stored on a microcomputer for off-line analysis. The number of action potentials/movement was determined using Spike 2 software (Cambridge Electronic Design, Cambridge, UK).

### Behavioral assessment of pain

For measuring joint pain behavior, MIA-treated rats were regularly handled and gradually habituated to the test equipment on three consecutive days prior to behavioral testing. Hindlimb weight bearing was determined using an incapacitance tester (Linton, Norfolk, UK) consisting of a dual channel weight averager. The force exerted by each hindlimb (measured in grams) was averaged over a five second period. Each data point is the mean of three consecutive readings. For MIA animals, weight distribution was measured between treated (intra-articular injection of A-803467 10 mg; 100 μl bolus) and contralateral non-treated hindlimbs. For secondary allodynia measurements, a von Frey hair algesiometer (Ugo-Basile, Milan, Italy) was employed to determine the force required to elicit a withdrawal response to a tactile mechanical stimulus applied to the hindpaw. The rat was placed on a metal mesh surface in an enclosed area and allowed to move about freely. With the animal at rest (that is, in the absence of any exploratory or grooming behavior) a touch stimulator unit was oriented under the animal and using an adjustable angled mirror the stimulating microfilament (0.5 mm diameter) was positioned below the plantar surface of the paw. Activation of the unit caused the fine metal monofilament to advance with constant speed and touch the paw in the proximal metatarsal region. The filament exerts a gradually increasing force to the plantar surface, starting below the threshold of detection and increasing until the stimulus becomes painful and the rat withdraws its paw. The force required to elicit a paw withdrawal reflex is automatically recorded and measured in grams. A maximum force of 50 g and a ramp speed of 4.5 g/second were chosen for all of the algesiometry tests.

### Drugs and reagents

Sodium monoiodoacetate and A-803467 were obtained from Tocris Bioscience (Minneapolis, MN, USA; Bristol, UK); gallamine triethiodide, DMSO, cremophor and urethane were obtained from Sigma Aldrich (Oakville, Ontario, Canada; Poole, UK). A-803467 was dissolved in vehicle solution (2% DMSO, 1% cremophor, 0.9% saline) and aliquots of the drug were kept frozen (-20°C) in Eppendorf vials until required. The pH of all solutions was determined to exclude acidity as a sensitizing factor on afferent nerve fibers. All solutions were found to have a neutral pH (pH 7.4) before injection. Gallamine triethiodide was made fresh on the day of experimentation and dissolved in 0.9% saline.

### Statistics

All data were normally distributed and expressed as means ± SEM for 'n' observations. Establishment of joint pain and secondary allodynia after MIA injection was confirmed using a Student's t-test. The effect of A-803467 on nociceptive activity was analyzed by two-way analysis of variance (ANOVA) with a Bonferroni post test. All differences were considered statistically significant when *P *was less than 0.05.

## Results

### Electrophysiological recordings

Between one and three afferent fibers were examined per animal such that a total of 19 units were recorded in this study. All fibers responded to noxious stimuli while only six units responded in both the normal and noxious working range. A wash out period of at least 60 minutes was observed between administration of A-803467 to multiple fibers in the same animal. In case the firing rate of a recorded afferent did not return to baseline level after 30 minutes no further afferents were recorded in that particular animal. The conduction velocities of recorded afferents ranged from 0.57 to 2.87 m/s. Approximately 20% of the recorded fibers were classified as Aδ fibers with the remaining fibers being slowly conducting C-fibers. The mechanical threshold of the recorded fibers ranged from 5 to 35 mNm The average firing rate of afferents in the MIA-injected joints during non-noxious joint rotation was 43 ± 6.21 action potentials/10 s movement (*n *= 10) and 114 ± 9.95 action potentials/10 s movement with noxious joint rotation (*n *= 16) These firing rates are consistent with previous studies that showed a significant increase in firing rate in MIA-treated rats compared to saline injected control joints [12].

### Effect of A-803467 on joint afferent mechanosensitivity

**Figure 1:**
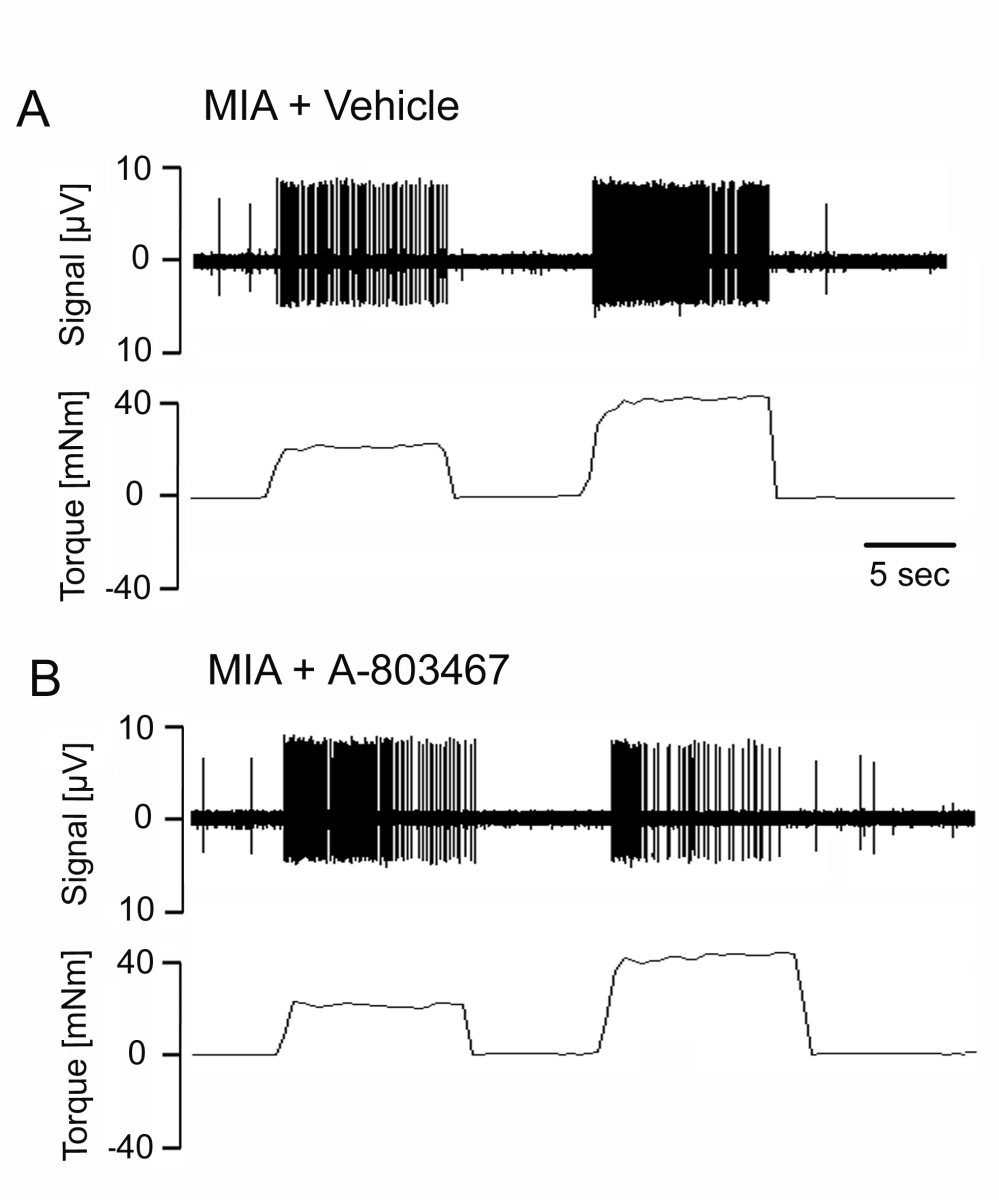
**Effect of Nav1.8 ion channel blockade on joint nociception**. Figure shows a typical single unit recording of an unmyelinated C-fiber (conduction velocity: 1.38 m/sec) in response to normal and noxious rotation of MIA arthritic knee joints. Recording shows nerve firing activity before **(A) **and after **(B) **close intra-arterial injection of A-803467. Coincidental torque levels are displayed below each nerve recording. Movement-evoked afferent firing rate was reduced by local administration of the Nav1.8 ion channel blocker.

**Figure 2:**
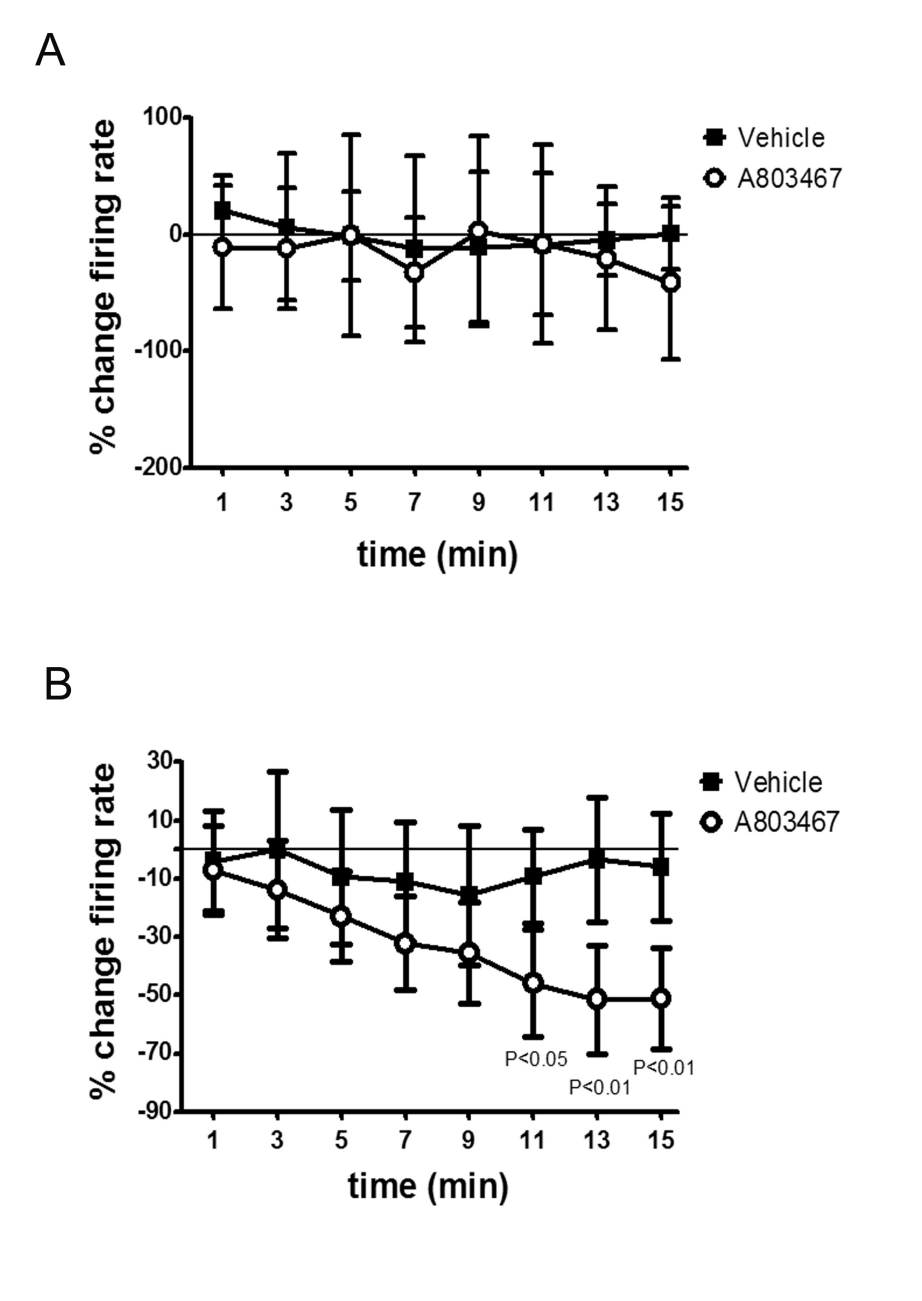
**Time course of effect of A-803467 on joint mechanosensitivity**. Local application of A-803467 had no effect on joint afferent firing rate during non noxious rotation of the joint (**A**) *n *= 6/group, but significantly reduced nociceptor activity during noxious joint rotation (**B**). 2-way ANOVA; *n *= 10 to12 vehicle group, *n *= 18 to 19 A-803467 group. Values are means ± SEM.

**Figure 3:**
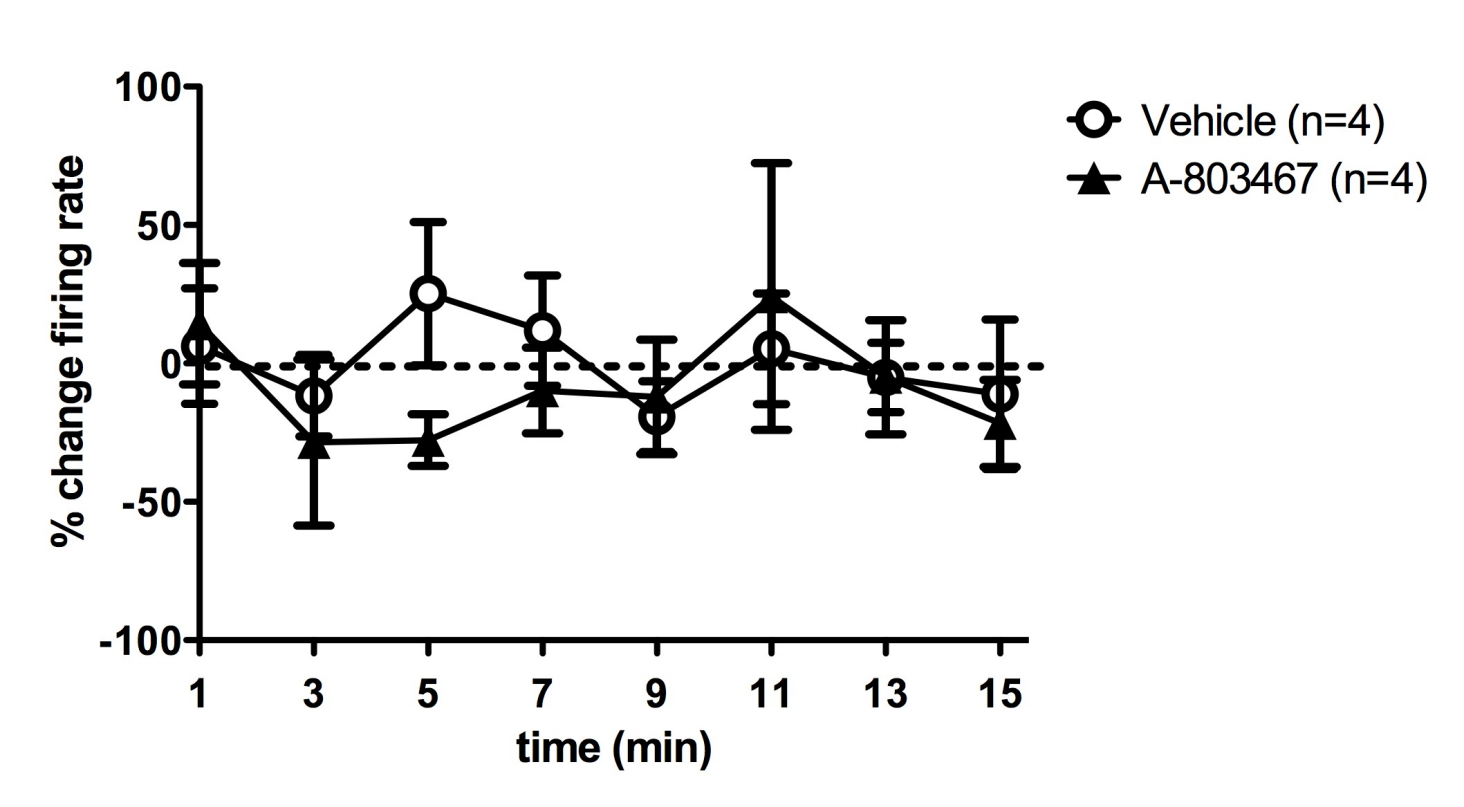
**Effect of A-803467 on joint afferent spontaneous activity**. Compared to vehicle, local injection of A-803467 had no effect on nerve spontaneous activity (*n *= 4/group). Values are means ± SEM.

With non-noxious rotation of the MIA-injected rat knee joint, local administration of A-803467 had no effect on mechanosensitivity of joint afferents; however, A-803467 reduced the mechanosensitivity during noxious rotation of the joint. A specimen recording from a typical joint afferent before and after either vehicle or A-803467 application is shown in Figure 1A and 1B respectively. The desensitizing effect of A-803467 during noxious joint rotation was significant at 11 minutes after drug application and reached a maximum at the 13 minute time point (mean: -51.45% ± 8.81; % change compared to control). Compared to vehicle, A-803467 had no significant effect on firing rate during non-noxious rotation (*P *= 0.49 two-way ANOVA with Bonferroni's post-test; *n *= 6/group (Figure 2A); but significantly reduced the firing rate during noxious joint rotation (*P *< 0.0001 two-way ANOVA with Bonferroni 's post-test; *n *= 10 to 12 vehicle group, *n *= 18 to 19 A-803467 group; Figure 2B). Both, A-δ fibers and C-fibers were equally responsive to A-803467. The vehicle was found to have no significant effect on joint mechanosensitivity. Spontaneous activity was observed in four of the recorded joint afferents. The mean frequency of spontaneous firing before drug application was 0.53 ± 0.1 Hz and 1 minute after A-803467 afferent activity was 0.63 ± 0.2 Hz. The lack of effect of A-803467 on spontaneous activity was consistent across the 15 minute time course perineurium (*P *= 0.46 two-way ANOVA; *n *= 4/group; Figure 3).

### Effect of A-803467 on joint pain and secondary allodynia

**Figure 4:**
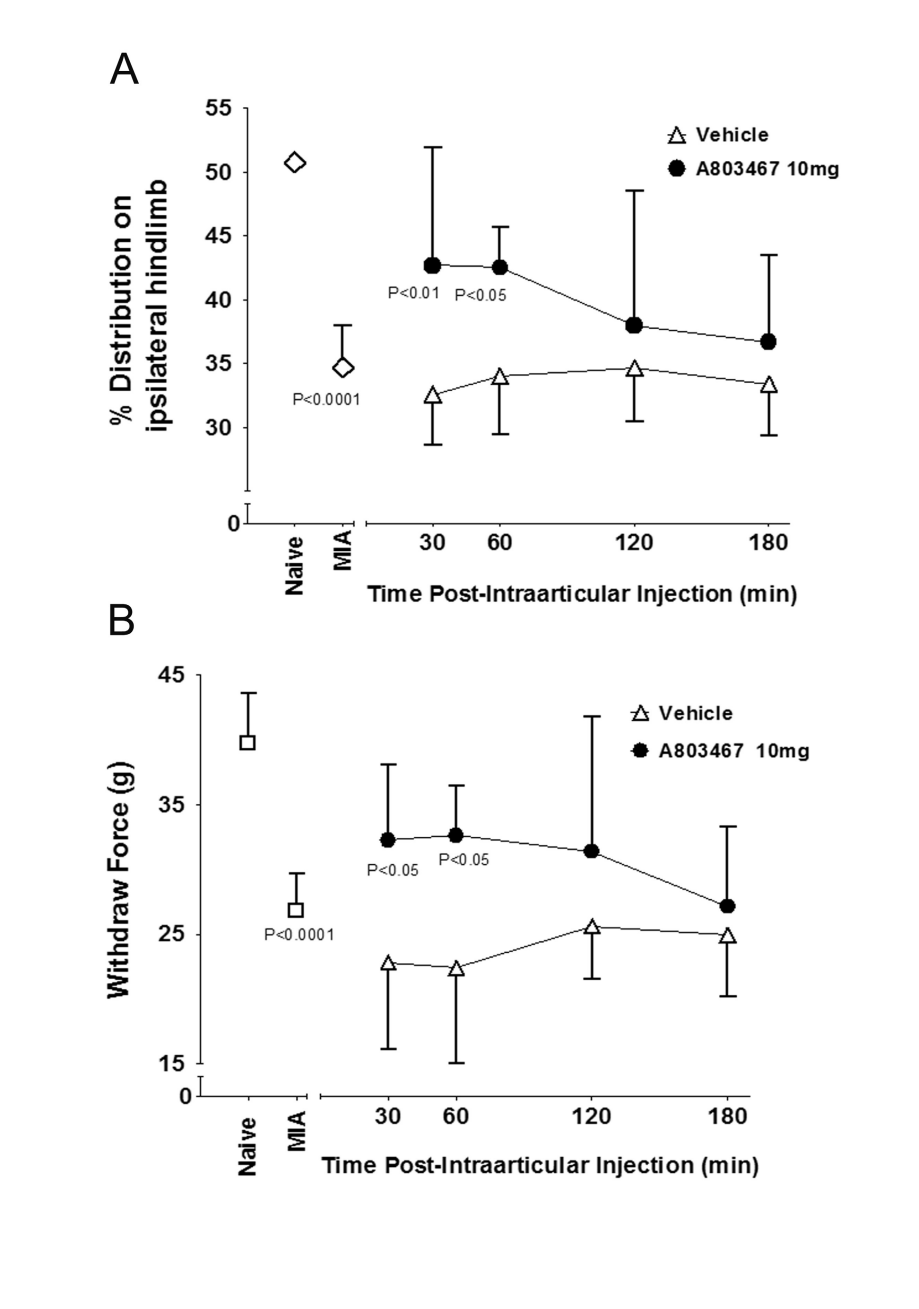
**Pain behavioral responses to treatment with A-803467**. In **(A)**, MIA causes a profound shift in hindlimb weight distribution onto the unaffected hindlimb. Following A-803467 treatment body weight was re-distributed onto the MIA-treated joint reflecting an analgesic effect. In **(B)**, hindpaw withdrawal thresholds were measured in response to an intra-articular injection of A-803467. The force required to elicit hindpaw withdrawal following A-803467 was significantly reduced compared to vehicle-treated animals, confirming a reduction in MIA induced secondary allodynia. Values are means ± SEM.

The induction of OA caused a significant reduction in the amount of weight being borne on the ipsilateral hindlimb compared to naïve rats (*P *< 0.0001; unpaired Student's t-test; *n *= 20 naïve rats, *n *= 6 OA rats; Figure 4A). While naive rats distribute their body weight evenly between the two hindlimbs, in the MIA rat the weight placed on the ipsilateral hindlimb was reduced to about 35% ± 1.46. Intraarticular injection of A-803467 into the MIA-treated joint reduced the difference in weight bearing significantly such that 43% ± 2.89 of the animal's body weight was now placed on the ipsilateral hindlimb. This improvement in weight distribution was significant compared to vehicle treated animals (*P *< 0.0001; two-way ANOVA with Bonferroni 's post test; *n *= 10; Figure 4A) indicating that A-803467 reduced pain in MIA joints. Injection of the vehicle by itself had no effect on weight distribution.

Paw withdrawal threshold to mechanical stimuli in naïve animals (mean: 39.7 g ± 1.69), was significantly reduced in MIA arthritic rats (mean: 26.80 g ± 1.27 indicating the presence of secondary allodynia (*P *= 0.0002; unpaired Student's t test; *n *= 10 naïve rats, *n *= 6 OA rats; Figure 4B). Intra-articular A-803467 injection significantly reduced this MIA-induced mechanical hypersensitivity (mean: 32.25 g ± 1.84) compared to vehicle treated animals (mean: 22.83 g ± 2.62; *P *< 0.0001, two-way ANOVA with Bonferroni's post test; *n *= 10; Figure 4B.

## Discussion

Management of osteoarthritis pain remains a huge challenge in the clinic with millions of arthritis patients living with chronic disability. Limitations to current pharmacological treatment strategies include a lack of therapeutic efficacy and potentially hazardous side-effects. Therefore, there is a pressing need to understand the underlying mechanisms that evoke OA pain and to develop new, more effective analgesics. One intuitive approach would be to attenuate pathological OA-induced pain by selectively blocking joint nociceptors while preserving other desirable physiological functions of the nerve. Intra-articular injection of non-selective sodium channel blockers has been shown to be effective for the treatment of OA pain [29] while intravenous administration of these agents can produce long lasting pain relief in both animal models [30] and in patients with intractable neuropathic pain [31]. The clear disadvantages of using non-selective sodium channel blockers locally are complete loss of sensation and disruption of normal physiological functions such as proprioception and neurovasoregulation. Systemic use of such blockers can lead to cardiotoxicity, sedation and cognitive impairment [32]. Targeting sodium channels which are located exclusively on nociceptive afferents could reduce these undesirable side effects. The novel small molecule A-803467 selectively blocks the Na_v_1.8 sodium channel *in vitro *and *in vivo *[25]. The agent has been shown to inhibit inward currents in cultured rat dorsal root ganglia cells and Na_v_1.8 expressing HEK cells (IC50 = 8 nM). A-803467 was also found to reduce mechanically-evoked firing of dorsal horn wide dynamic neurons as well as mechanical allodynia in animal models of inflammation and neuropathic pain over the dose range 10 to 100 mg/kg. In this study it was found that local administration of A-803467 significantly reduced OA joint mechanosensitivity during noxious but not during non-noxious rotation of the joint. These data confirm that blocking the Nav 1.8 channel attenuates joint mechanonociception in the MIA model of joint degeneration. The fact that the reduction in mechanosensitivity was limited to noxious joint rotation confirms that Nav1.8 plays a major role in transmitting strong noxious signals but does not affect physiological mechanosensitivity during normal, non-noxious rotation of the joint.

Approximately 50% of C-fibers and 10% of A-δ fibers in naive rats express Nav1.8 channels [33] and this proportion increases in painful conditions [21,34]. In addition, a dynamic redistribution of Nav1.8 channels from dorsal root ganglia somata to the axons has been observed so that more Nav1.8 channels are expressed on C-fibers and A-δ fibers in rat digital nerves after neuropathic injury [35]. In this study, the firing rate in about 75% of all recorded afferents was substantially reduced during noxious rotation after A-803467 administration. The relatively high percentage of responsive fibers to A-803467 in this study could be explained by similar channel reorganization and up-regulation in response to MIA induced joint pathology. Systemic administration of A-803467 has been shown to reduce pain significantly in several models of neuropathic and inflammatory pain [25-27] while preserving motor function as assessed by spontaneous exploratory behavior, and motor coordination as assessed by the rotarod and edge test assays [25]. In this study, an intra-articular injection of A-803467 significantly reduced hindlimb incapacitance and secondary allodynia in MIA-treated rats. This observation corroborates the electrophysiological data which signified an anti-nociceptive effect of the channel blocker in diseased joints. The inhibitory effect of intra-articular A-803467 on hindlimb incapacitance confirms that joint Nav1.8 channels are involved in OA pain and that peripheral modulation of these channels has analgesic potential. The ability of intra-articular A-803467 to reduce secondary allodynia in this study is also of importance as it suggests that the referred pain associated with OA can be moderated by blocking Nav1.8 channels in the knee.

Although the evidence presented here highlights the involvement of peripheral Nav1.8 channels in producing OA pain, Nav1.8 channels present on the central terminals of primary afferent nerves also need to be considered. It has recently been demonstrated that A-803467 has good penetration into the central nervous system where it produces a more robust antinociception in neuropathic pain models than a peripherally acting non-selective sodium channel blocker [36]. This ability of A-803467 to suppress spinal dorsal horn neuronal excitability and to reduce allodynia effectively after nerve injury provides further evidence that inhibition of Nav1.8 channels on peripheral nerves, with synaptic connections in the spinal cord, is an important site of nociceptive sensory processing [37] and could be necessary to achieve more pronounced and longer lasting analgesia.

The present investigation also found that A-803467 had no significant effect on spontaneous nerve activity in MIA-treated joints. The reason for the lack of effect of peripheral A-803467 administration on MIA-induced spontaneous activity could be that articular Nav1.8 channels are only activated by external physical stimuli and are unaffected by the sensitizing effect of algogenic agents present in the diseased joint. The central terminal of the primary afferent neuron, however, could still be a useful target for reducing spontaneous activity and could therefore play a role in the reduction of OA pain at rest. This hypothesis is supported by studies showing that intrathecal administration of A-803467 significantly reduced evoked and spontaneous firing of wide dynamic range neurons [25] whereas local intra-plantar application only reduced evoked but not spontaneous nerve firing [27]. Since spontaneous activity is necessary for the perception of chronic, on-going pain in MIA joints [11], intrathecal A-803467 may be required to address the problem of pain at rest in OA patients.

In conclusion, this study shows for the first time that the Nav1.8 channel is involved in mediating nociception and pain in a rat model of OA. We propose that targeting Nav1.8 channels could be a promising approach to reduce nociceptor sensitivity and consequently pain perception in OA while avoiding the undesirable side-effects of non-selective sodium channel blockers.

## Abbreviations

ANOVA: analysis of variance; MIA: monoiodoacetate; OA: osteoarthritis; VGSC: voltage gated sodium channel.

## Competing interests

The authors declare that they have no competing interests.

## Authors' contributions

NS helped design the experiments, carried out the electrophysiology, helped analyze the data and contributed to writing the manuscript. JJMcD helped design the experiments, assisted in data analysis and helped write the manuscript. Both authors read and approved the final manuscript.
